# The peptide genomic therapy increases antibacterial immunity and survival in sepsis by reprograming the gene orthologs of human immunodeficiencies in the spleen and lungs

**DOI:** 10.3389/fimmu.2025.1635081

**Published:** 2025-10-28

**Authors:** Huan Qiao, Jozef Zienkiewicz, Yan Liu, Jacek Hawiger

**Affiliations:** ^1^ Vanderbilt University School of Medicine, Department of Medicine, Division of Allergy, Pulmonary and Critical Care Medicine, Nashville, TN, United States; ^2^ Department of Veterans Affairs, Tennessee Valley Health Care System, Nashville, TN, United States; ^3^ Vanderbilt University School of Medicine, Department of Molecular Physiology and Biophysics, Nashville, TN, United States

**Keywords:** inborn errors of immunity (IEI), nuclear transport checkpoint inhibitor (NTCI), polymicrobial sepsis, cSN50.1, cell-penetrating peptides, inflammatory regulome, gene expression, RNAseq

## Abstract

Sepsis is a life-threatening complication of infections afflicting 49 million patients worldwide with 11 million sepsis-related deaths. In the USA, this growing public health problem concerns 1.7 million adult and pediatric patients. An estimated 1 million patients with asplenia or hyposplenia are particularly vulnerable to sepsis. We show that the Peptide Genomic Therapy (PGT) with the cell-penetrating Nuclear Transport Checkpoint Inhibitor (NTCI) peptide increased 29 times bacterial clearance in the spleen, a major blood-filtering immune organ in the preclinical model of sepsis, when combined with the antibiotic. Likewise, the PGT with NTCI peptide increased antibacterial immunity in the lungs, the frequent site of bacterial infections in spleen-compromised hosts. The survival reached 80% when the NTCI peptide was added to antibiotic, compared to 44% with meropenem alone. The NTCI peptide reprogrammed the expression of the gene orthologs responsible for human immunodeficiencies, also referred to as the Inborn Errors of Immunity (IEI). The 227 IEI genes were reprogrammed in the spleen and 215 in the lungs, while the mediators of inflammation in blood (IL-6, IL-10, TNFα, Interferon γ, and MCP1) were normalized by the NTCI peptide. Thus, the PGT with NTCI peptide combined with antibiotic significantly increased the antibacterial immunity in the spleen and lungs and almost doubled survival in sepsis.

## Introduction

Sepsis, a life-threatening stage of multi-organ microbial inflammation caused by bacteria, viruses, fungi and protozoa, is alarmingly growing worldwide, as almost 49 million sepsis patients with 11 million sepsis-related deaths were reported in 2017 (1). In the USA, at least 1.7 million pediatric and adult patients develop sepsis each year, and 350,000 die of the illness (2). Thus, one in three patients who died in the USA hospitals was diagnosed with sepsis. Sepsis is mediated by uncontrolled microbial inflammation (3). The proinflammatory effectors of innate and adaptive immunity (cytokines, chemokines, and acute phase proteins), contribute to this life-threatening complication of spreading infection (4).

Estimated 1 million immunocompromised patients with asplenia or hyposplenia in the USA face a high risk of sepsis (5). This risk attests to the protective function of the spleen as the major blood-filtering immune organ. Therefore, the asplenic and hyposplenic patients are particularly vulnerable to the invasive infections by *Streptococcus pneumoniae*, *Neisseria meningitides, and Hemophilus influenzae*, among others. Their initial entry into the lungs and uncontrolled spread to other organs (e.g., central nervous system) leads to the potentially lethal sepsis (6, 7).

The innate and adaptive immunity in the spleen and other lymphoid organs is programmed by the hundreds of immune response genes that encode the cellular and humoral effectors. The immunoprotective function of these genes was revealed by the “experiments of nature”, known as the Inborn Errors of Immunity (IEI). Hence, the children and adults with the IEI are highly vulnerable to the recurrent bacterial, viral, and fungal infections potentially evolving into sepsis. The IEI have led to the discovery of at least 485 human genes linked to the clinically defined immunodeficiencies consisting of the well-characterized monogenic immune diseases (8). For example, the IEI genes underly the recurrent severe and opportunistic infections in combined immunodeficiency that includes the genes encoding CD40, CD40 ligand (CD145), ICOS, ICOS ligand (ICOS L), CD8, MHC Class I and MHC Class II, IKAROS, Polymerase δ, LCK, MALT1, CARD11, BCL10, IL-21, and IL21R. The products of these genes are harbored mainly in the spleen, other lymphoid organs, bone marrow, liver, and blood. Thus, the individuals who lack spleen (asplenia) or have functional insufficiency of the spleen (dysplenia) due to the Sickle Cell Disease, Celiac Disease, or bone marrow transplantation (7, 9), are particularly vulnerable to the life-threatening invasive infections evolving into sepsis.

The vulnerability of patients with asplenia or dysplenia to sepsis prompted us to formulate “the IEI genes in sepsis are regulated by the NTCI peptide” hypothesis. We posited that during sepsis some IEI genes are upregulated, downregulated or unchanged. Further, we also postulated that these responses of the IEI genes during sepsis will be modified by the Peptide Genomic Therapy (PGT) with the Nuclear Transport Checkpoint Inhibitor (NTCI) peptide. We focused on the response of the 485 IEI gene orthologs in the spleen and lungs during the polymicrobial sepsis in an experimental model. This preclinical model is relevant to human sepsis as humans and mice show 85% similarity of their DNA sequences that code for proteins (10) (National Human Genome Research Institute).

We designed and studied the cell-penetrating NTCI peptides to control the signal transduction and gene transcription underlying inflammatory response. These signaling pathways include the families of stress-responsive transcription factors, SRTFs (e.g., NF-κB, cFos, cJun, STAT1 and STAT3, NFAT, and Nrf2), and Metabolic Transcription Factors, MTFs (SREBPs and ChREBPs) (11–15). Their genomic targets encode inflammatory mediators, cytokines and chemokines, among others, as well as effectors of the metabolic synthetic pathways (13, 16). The access of SRTFs and MTFs, to the inflammatory and metabolic regulomes in the genome depends on the nuclear transport checkpoint, comprising Importin α5 and Importin β1, among others (16). Importin α5, aka karyopherin α1, is one of the six members of the importin α family (17). Importin α5 recognizes Nuclear Localization Sequence (NLS) on multiple proinflammatory SRTFs and metabolic transcription factors, ChREBPs (18). Importin β1 selectively recognizes SREBPs (19). These proinflammatory and metabolic transcription factors regulate a myriad of genes that encode mediators of microbial and metabolic inflammation (16). The NTCI peptide targets two nuclear transport shuttles and blocks the nuclear signaling responsible for gene activation in both inflammatory and metabolic pathways. The NTCI peptide comprises the cell-membrane translocating motif and NLS. The NTCI peptide is biselective that binds to both importin α5 and Importin β1 (20). We posited that the PGT with NTCI peptide controls expression of proinflammatory and metabolic genes in sepsis.

We found that survival reached 80% in the polymicrobial sepsis model when the PGT with NTCI peptide was combined with the antibiotic (Meropenem), which alone afforded survival only at the 44% rate. The NTCI peptide also improved hypothermia. These improvements were accompanied by changes in the expression of the IEI gene orthologs: 102 in the spleen and 94 in the lungs out of the 485 known IEI genes. Among them, 40 IEI genes were enhanced and 62 were suppressed in the spleen. Accordingly, 12 IEI genes were upregulated, and 82 genes were downregulated in the lungs. Markedly, the IEI genes that encode the inflammatory mediators in blood (cytokines IL-6, IL-10, TNFα, Interferon γ, and chemokine MCP1) were suppressed by the PGT with the NTCI peptide. These mediators contribute to organs injury caused by the virulence factors of invading microorganisms during the polymicrobial sepsis. Thus, the reprogramming of the IEI genes in the spleen and lungs together with the suppression of the key mediators of inflammation in the blood by the NTCI peptide, contributed to the remarkable improvement in sepsis survival.

## Results

### Survival and hypothermia in sepsis are improved by the peptide genomic therapy with NTCI peptide, added to antimicrobial therapy

**Figure 1:**
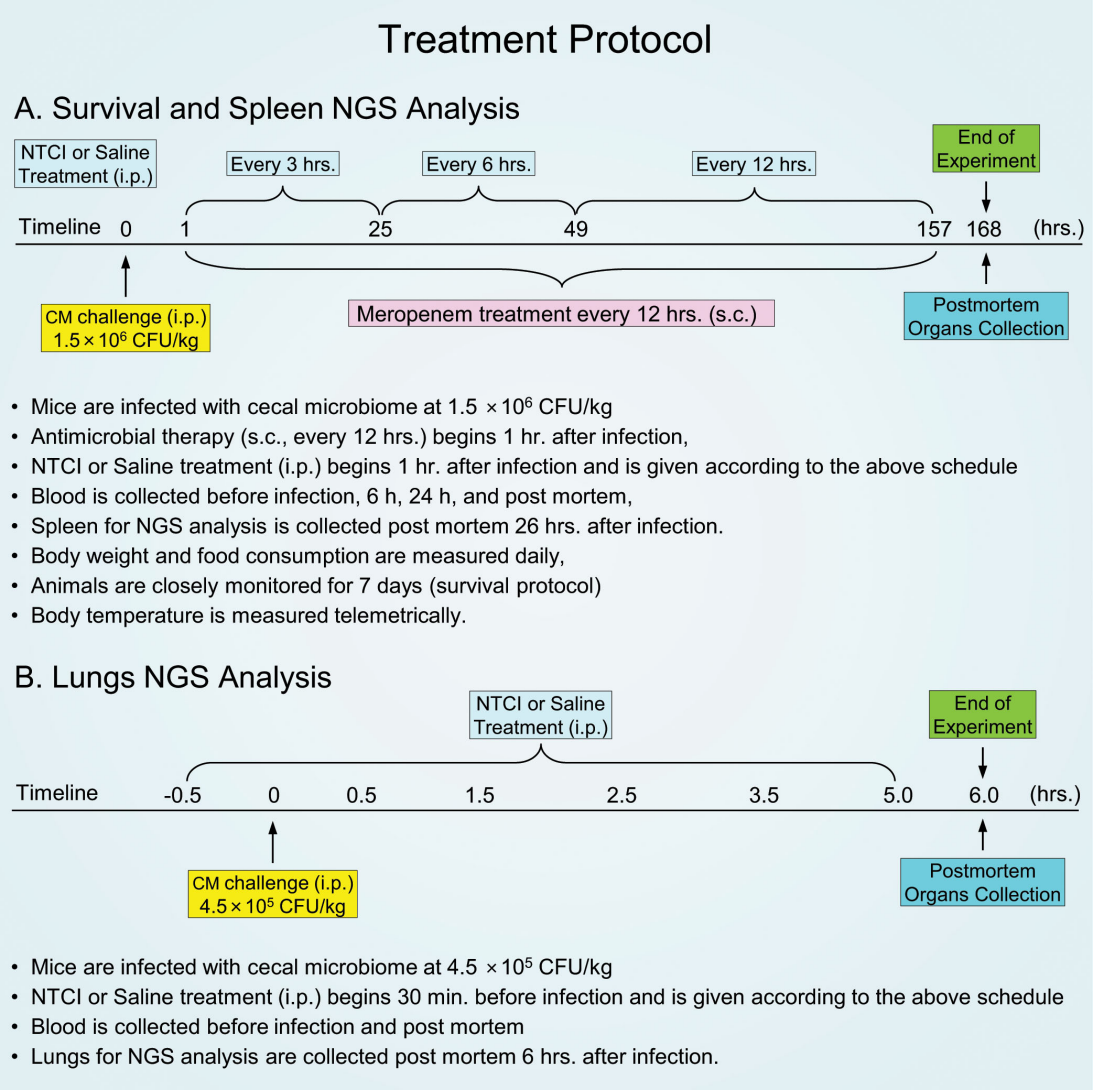
Graphic depiction of two treatment protocols. **(A)** Survival treatment protocol: 8-week-old C57BL/6 female mice were infected with i.p. injection of CM (1.5×10^6^ CFU/kg) and treated with saline, meropenem with saline or meropenem with NTCI peptide for for 7 days. **(B)** Short treatment protocol for lungs NGS analysis: 8-week-old C57BL/6 female mice were infected with i.p. injection of CM (4.5×10^5^ CFU/kg) and treated with saline or with NTCI peptide for 5 hours.

To study the anti-bacterial immunity in the spleen and lungs, we adopted the clinically relevant experimental model of sepsis evolving from polymicrobial peritonitis. This massive peritoneal infection causes sepsis that provides consistent results based on the standardized microbial challenge as compared to the alternative surgical model, the cecal ligation and puncture. The latter produces uncontrolled spillage of the cecal microbiome into the peritoneal cavity. We obtained the cecal microbiome (CM), aka “cecal slurry”, from the euthanized healthy donor mice (15). Of note, the gut microbiome in these C57Bl/6 mice comprises Dorea, Actinobacteria, Bifidobacteria, Turucibacteriaceae, Firmicutes, Lactobacilli, and other Bacilli (21). Animals were challenged with the titrated amount of gut microbiota through a single intraperitoneal injection of CM [1.5×10^6^ CFU (colony forming units) per kilogram] (**Figure 1**) representing LD_60_ determined in the antibiotic-treated group (**Supplementary Figure S1**). Thus, the mice were uniformly infected with the precisely quantified CFUs. We stress that this microbial challenge comprises a multitude of intestinal Gram-negative bacteria expressing diverse virulence factors, e.g. Lipopolysaccharides (LPS), Gram-positive bacteria, and other microbes residing in the gut microbiome (15, 22). The resulting polymicrobial peritonitis is accompanied by the dissemination of the infection to the blood (bacteremia), spleen, lungs, kidneys, and other organs (4). This mechanism of sepsis is akin to human sepsis evolving from the infected single organ due to microbial spread culminating in a multi-system microbial inflammation (16).

The CM-infected animals were subjected to fluid resuscitation (0.45% NaCl, 200 μl) therapy following the treatment protocol presented in **Figure 1**. Therein, this group is described as CM + Saline. Another experimental group comprised CM-infected animals treated with fluid resuscitation and antimicrobial therapy with antibiotic meropenem (25 mg/kg administered s.c.), which began 1h after infection with CM and continued every 12 hrs. until euthanasia. This group is denoted as CM + Mero. The third experimental group comprised CM-infected animals treated with combined therapies of fluid resuscitation, antibiotic, and the PGT with NTCI peptide (cSN50.1) at dose of 33 μg/g/injection in 200 μl 0.45% NaCl). This group is depicted as CM + Mero + NTCI in the figures. The NTCI peptide (cSN50.1) was selected for its efficacy as the strongest inhibitor of inflammatory responses in the murine models of microbial, metabolic, and allergic inflammation (4, 13, 14, 18, 23, 24). This cell-penetrating peptide targets importin α5 (Imp α5, IPOA5, KPNA1), a mediator of nuclear import of stress-responsive transcription factors families such as NF-κB, NFAT, STAT, AP-1 and NRF2 (16). As a background of gene expression used for comparison in NGS analysis, the animals were sham-infected (5% dextrose) and treated with fluid resuscitation.

**Figure 2:**
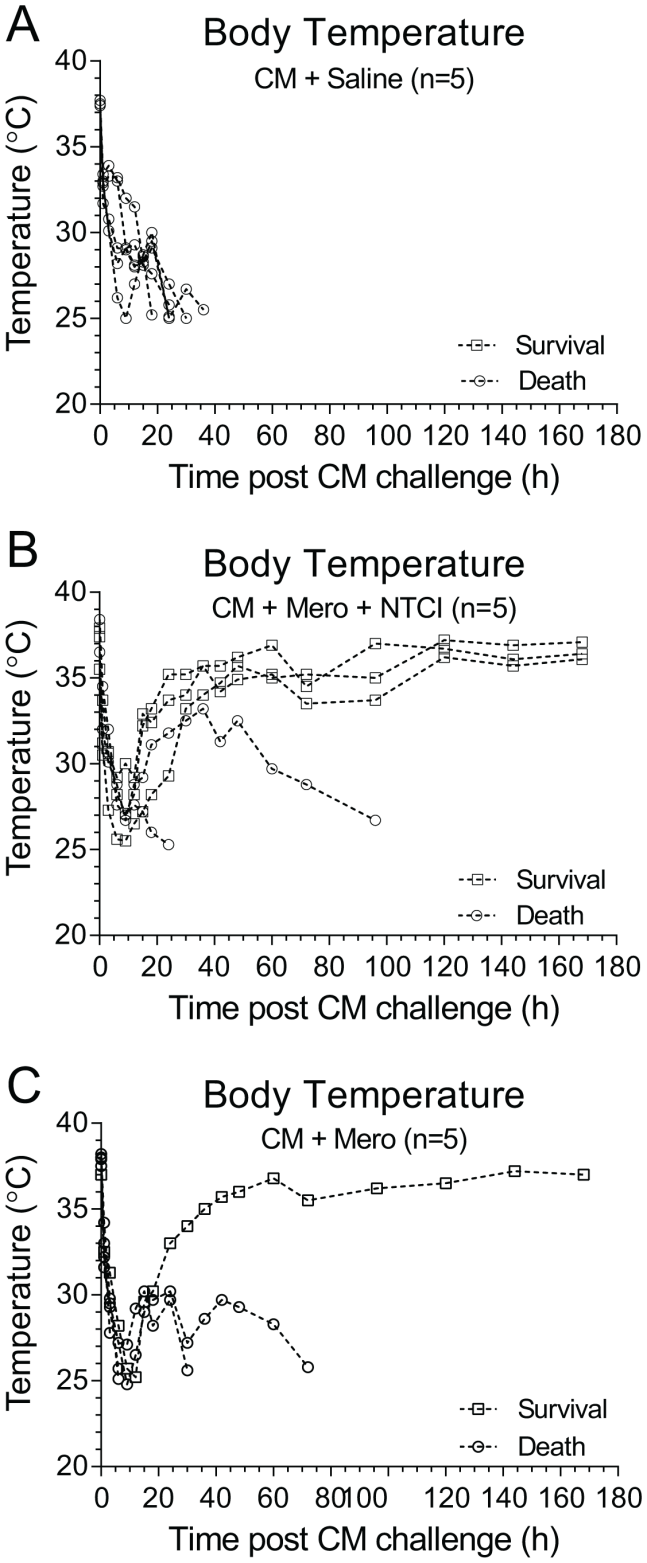
Severe hypothermia in CM-infected mice is improved by peptide genomic therapy with NTCI peptide. Sepsis-induced sudden drop in body core temperature is corrected with PGT by NTCI peptide. Body temperature was measured wirelessly with subcutaneously implanted RFID temperature transponder (IPTT-300, see Materials and Methods for details).

**Figure 3:**
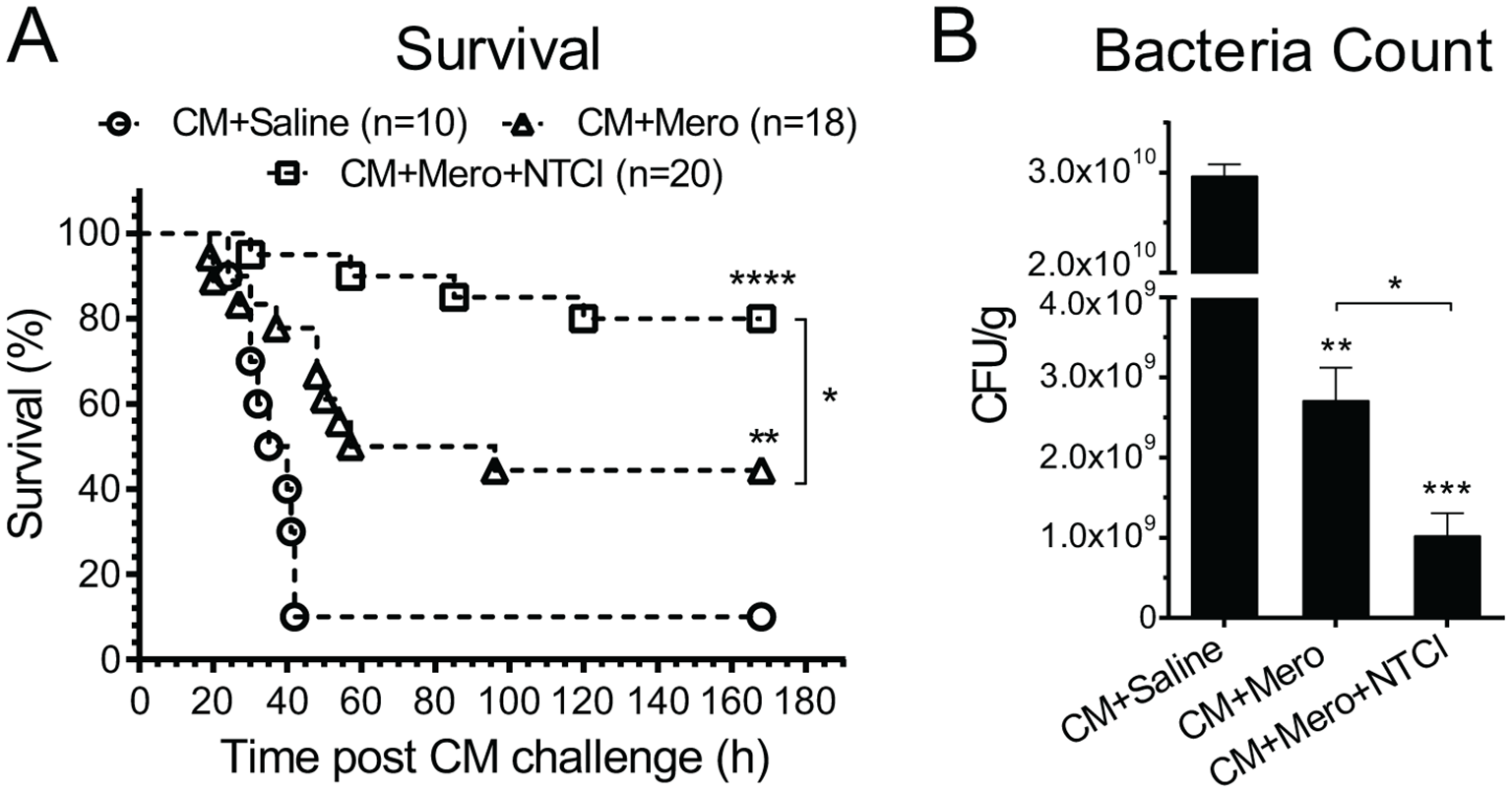
Peptide genomic therapy with NTCI improved survival and reduced bacterial dissemination in spleen of CM-infected mice. Mice with CM-induced (i.p.) polymicrobial peritonitis were treated for 7 days with the saline, meropenem, or with meropenem and NTCI (see Materials and Methods for details). **(A)** Treatment with NTCI peptide significantly improved survival (80%, median survival undefined, mean time-to-death 73 hrs., hazard ratio vs. Saline 0.11) of CM-infected mice as compared to Meropenem (44%, median survival 76.5 hrs., mean time-to-death 45.6 hrs., hazard ratio vs. Saline 0.33) and saline (10%, median survival 37.5 hrs. mean time-to-death 35.1 hrs.). Data is presented as Kaplan–Meier survival plot with p value determined by log rank analysis, *p < 0.05, **p < 0.005, ****p < 0.0001. **(B)** Mice were euthanized 26 hrs. post CM infection and spleen was collected and processed for bacterial count (n=5) (see Materials and Methods for details). Bacterial dissemination was analyzed by nonparametric t test with Mann–Whitney rank comparison. The data is presented as a mean ± SEM. *p < 0.05 **p < 0.005, ***p < 0.0005.

We recorded a precipitous drop in body temperature from 38°C to 32°C within 1 hour after the onset of the polymicrobial sepsis in adult C57Bl/6 mice (**Figure 2**). Of note, such a decrease in body temperature (hypothermia) is usually associated with a high mortality rate in serious bacterial infections of infants ≤90 days of age (25). The PGT with NTCI peptide combined with a broad-spectrum antibiotic, Meropenem, restored normal body temperature (**Figure 2C**) whereas treatment with antibiotic only partially reversed the hypothermia (**Figure 2B**). Most importantly, the survival reached 80% when the NTCI peptide was added to the antibiotic therapy (**Figure 3A**), compared to the 44% in the group treated with the antibiotic only. The mean time-to-death was similarly extended by the NTCI peptide (in treatment groups with Saline: 35.1 h, with Meropenem: 45.6 h, and with a combination of Meropenem and NTCI peptide: 73.0 h). Thus, the doubling of the time to death in the group treated with the NTCI combined with Meropenem paralleled the gain in the survival.

### Enhancement of anti-bacterial immunity in the spleen and lungs by the NTCI peptide in the polymicrobial sepsis

We postulated that the salutary action of PGT with the NTCI peptide on the survival in polymicrobial sepsis was linked to the enhancement of the anti-bacterial immunity in the spleen, lungs and other organs. Meropenem administered alone reduced 11-fold the bacterial count in the spleen indicating bacterial clearance therein. Addition of the NTCI peptide to the treatment protocol with Meropenem produced a 29-fold reduction of bacterial count, in the spleen of septic animals (**Figure 3B**). This impressive enhancement of bacterial clearance by the NTCI peptide was not due to its direct bactericidal activity toward cecal microbiome (4). Thus, the NTCI peptide combined with the antibiotic therapy enhanced the antibacterial immunity of the spleen. We analyzed the genomic mechanism of immunoenhancing action of the Peptide Genomic Therapy with NTCI peptide in the spleen and lungs (see below).

### Suppression of the major mediators of inflammatory response to sepsis in blood including IL-6, IL-10, TNF-α, IFN-γ, and a chemokine MCP1 (CCL2) by the peptide genomic therapy with NTCI peptide

**Figure 4:**
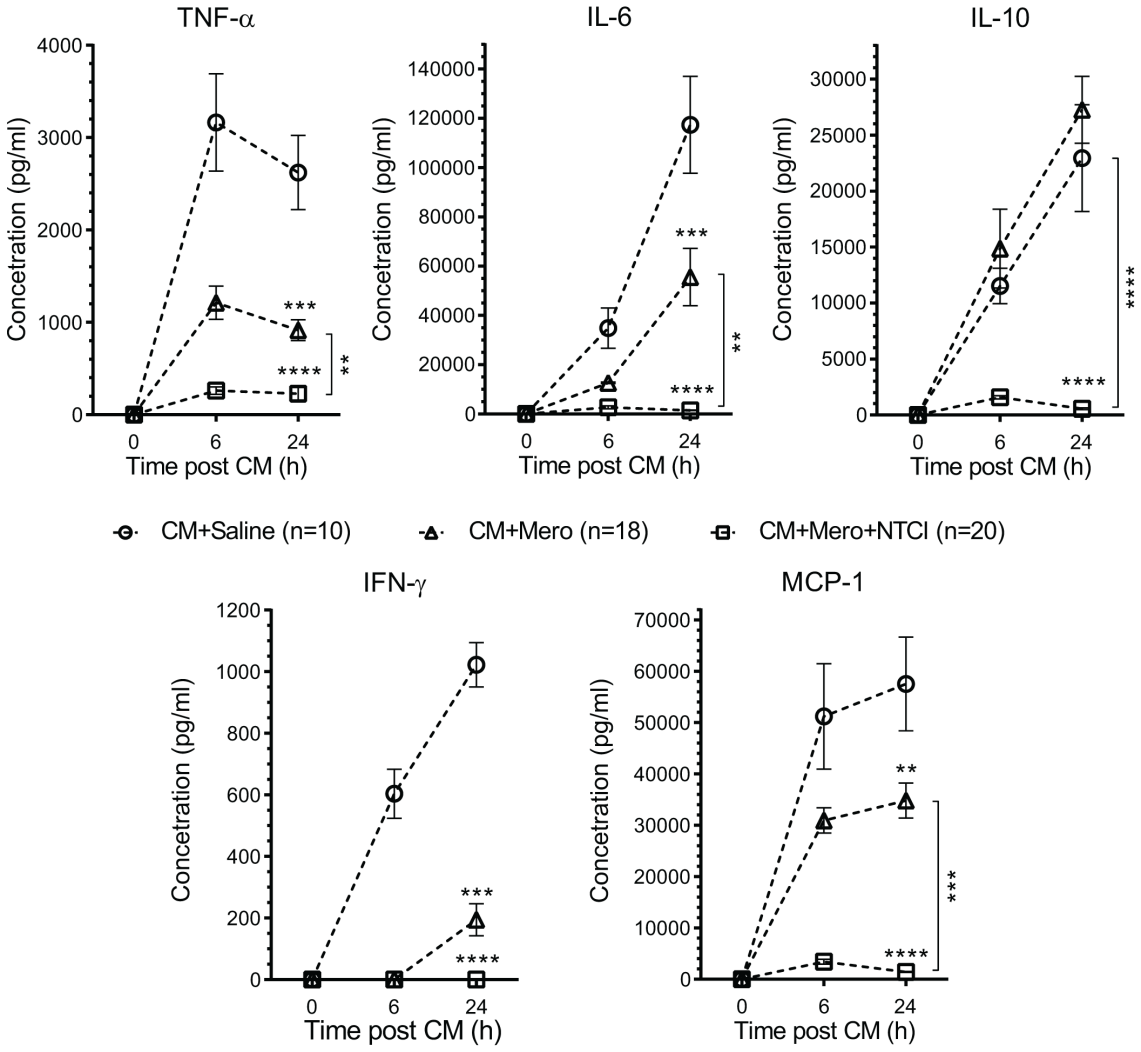
CM-induced production of mediators of inflammation is suppressed by peptide genomic therapy with NTCI. Cytokines (TNF-α, IL-6, IL-10, and IFN-γ) and chemokine MCP-1, levels were determined in blood plasma collected from saphenous vein before and 6, and 24 hrs. after CM challenge. The Peptide Genomic Therapy with NTCI significantly suppressed production of all analyzed mediators of inflammation. Data is presented as a mean ± SEM (n = 6). Statistical significance was determined by repeated measures two-way ANOVA analysis of variance with Holm-Sidak test for multiple comparison, **p < 0.005, ***p < 0.0005, ****p < 0.0001.

We monitored the time course of induction of the major mediators of inflammatory response to sepsis in blood by measuring the protein levels of 5 cytokines and 1 chemokine MCP1 (CCL2) in plasma (**Figure 4**). Antibiotic treatment partially reduced their plasma levels, with a notable exception of IL-10 (see below). The NTCI peptide added to the antibiotic lowered all inflammatory mediators to their baseline level, consistent with the significant gain in survival and enhancement of bacterial clearance in blood and lungs that underlies anti-bacterial immunity (see below).

### Reprogramming of the inborn errors of immunity (IEI) gene orthologs responding to the polymicrobial sepsis in the spleen and lungs by the Peptide Genomic Therapy with NTCI peptide.

We asked the question whether and how the expression profile of the human IEI gene orthologs in the spleen transcriptome correlates with the 29-fold enhancement of the bacterial clearance in the spleen by the PGT with NTCI peptide. The IEI gene orthologues encode a variety of the detectors of bacterial virulence factors, known as the pattern-recognition receptors, Toll-like Receptors (TLRs), other intracellular receptors, their adaptors, and signaling intermediates, including activated transcription factors and intracellular suppressors of cytokine signaling (16, 26). Among them, the innate immunity receptors, e.g., TLRs, are displayed not only on the immune cells but also on non-immune cells, including microvascular endothelial cells and epithelial cells (27). Therein, through their signaling intermediates, e.g. MyD88, and STING adaptors (28, 29), the nuclear signaling pathways are activated. These pathways include the families of stress-responsive transcription factors, SRTFs (e.g., NF-κB, cFos, cJun, STAT1 and STAT3, NFAT, and Nrf2), and Metabolic Transcription Factors, MTFs (SREBPs and ChREBPs) (11–15). The multitude of their genomic targets encode inflammatory mediators, cytokines and chemokines, among others, as well as effectors of the metabolic synthetic pathways (13, 16). The PGT with NTCI peptide blocks the nuclear signaling responsible for gene activation in both inflammatory and metabolic pathways.

The expression of IEI genes in the spleen was increased by Sepsis from 2- to 170-fold (see **Supplementary Table S1**). We highlight that the gene encoding IL-10 is the most sepsis-upregulated IEI gene (170-fold). Interleukin- 10 is a highly pleiotropic cytokine that potentiates the inflammatory response of activated CD8^+^ T-cells. The PGT with NTCI peptide reduced 6.5-fold the expression of *Il10* gene. IL-10-triggered signaling to the nucleus is mediated by the two stress-responsive transcription factors, STAT1 and STAT3 (30). We previously established that the NTCI peptide prevented the nuclear translocation of STAT1 and STAT3 to the nucleus (11, 15, 31). Hence, their target gene that encodes inflammatory mediator, Interferon (IFN) γ, among others, was not activated (see its low level in plasma during sepsis treated with the NTCI peptide) (**Figure 4**). Cumulatively, the PGT with NTCI peptide suppressed: (i) the expression of *Il10* gene, (ii) its elevated protein level in plasma, and (iii) its signaling intermediates in the spleen and other organs.

The other “top genes” induced by sepsis in the spleen encode Semaphorin 3e (*Sema3e*), Interleukin-17F (*Il17f*), and Cytotoxic T lymphocyte antigen 4 (*Ctla4*) (see **Supplementary Table S1**). *Sema3e*, aka Plexin D_1_, encodes a neural guidance molecule, which also impedes the migration of monocytes/macrophages and dendritic cells during the inflammatory response. Semaphorin 3e’s involvement in the airway’s pathobiology, such as asthma, has been reported (32). *Il17f* encodes one of the 6 members of the IL-17 family known to control bacterial and fungal infections (33). *Il17f* is also linked to chronic rheumatic immune diseases. We stress its extraordinarily high expression in the polymicrobial sepsis, as documented herein. This underscores the function of IL-17F as the top inflammatory mediator of uncontrolled bacterial infections evolving into sepsis. The NTCI peptide effectively suppressed genes encoding IL-17F and Sema 3e.

Another sepsis-upregulated gene *Ctla4* encodes CTLA4, the immune checkpoint that mediates immunosuppression. It is expressed on the lymphocytes and antigen-presenting cells (34). We emphasize that the remarkable 21-fold increase of *Ctla4* gene in the spleen during sepsis explains the immunosuppressive stage of this life-threatening complication. The Peptide Genomic Therapy with NTCI peptide reduced the elevated *Ctla4* gene expression by almost 5-fold during the polymicrobial sepsis. Such decisive genomic control opens an exciting approach to counteracting immunosuppression in the late stage of sepsis, thereby contributing to the enhancement of the anti-microbial immunity. Of interest, the gene encoding another checkpoint inhibitor, PD1, was not induced by sepsis in our study (see **Supplementary Table S1**). Others reported that an extracellular peptide targeting PD1 in the surgical model of sepsis increased survival (35). Furthermore, the PGT with NTCI peptide significantly suppressed sepsis-induced 5-fold increase of Interferon γ gene expression (see **Supplementary Table S1**). In parallel, the gene encoding Suppressor of Cytokine Signaling (SOCS1), that counteracts IFN-γ action (36), was reduced 2.5-fold by the NTCI. These examples support the NTCI peptide as a highly effective anti-inflammatory agent restoring and/or enhancing anti-microbial immunity in the spleen, lungs, and potentially, other organs during sepsis.

At the opposite end of the spectrum of IEI genes induced by sepsis, we found that the most reduced genes are those encoding *Nlrp12* (40x), *Cxcr2* (35x) and *Cr2* (12x). The Peptide Genomic Therapy with NTCI peptide reversed their suppression 4, 8, and 4 times, respectively (see **Supplementary Table S2**). NLRP12, the nucleotide-binding oligomerization domain-like receptor protein 12, is a member of the inflammasomes family, comprising intracellular sensors of virulence factors of phagocytized microorganisms causing cell stress (37). Thus, the 4-fold increase in the *Nlrp12* gene expression by the PGT with NTCI peptide adds to its restoration of the anti-bacterial innate immunity. In turn, sepsis-induced suppression of the gene encoding chemokine receptor CXCR2 was also reversed by the NTCI peptide. The *Cxcr2* gene is highly expressed in human and murine neutrophils. The CXCR2 ligands, including CXCL8, promote neutrophils migration to the inflamed liver and other organs to clear invading microbial agents (38). Finally, the NTCI peptide reversed the sepsis-induced suppression of the gene encoding the CR2 complement receptor that mediates complement activation through the alternative pathway (39).

## Discussion

The following new concepts concerning genomic mechanism of sepsis have emerged from our study. First, the survival almost doubled (from 44% to 80%) in the preclinical model of sepsis treated by the Peptide Genomic Therapy (PGT) with Nuclear Transport Checkpoint Inhibitor (NTCI) peptide. Hence, the NTCI peptide may be considered as the potential adjunct to the antimicrobial therapy that is routinely used in the management of patients diagnosed with sepsis in the USA (40). Second, the significant enhancement of the anti-bacterial immunity in the spleen and lungs by the PGT with NTCI peptide during the polymicrobial sepsis underscores the potential gain in survival. Third, the hitherto unknown response of the IEI gene orthologs in sepsis (102 in the spleen and 94 in the lungs out of the 485 known IEI genes), and their subsequent regulation by the NTCI peptide. Fourth, the identification of the IL-10, Semaphorin 3e, and IL-17F as the top responders to the polymicrobial sepsis that are being suppressed by the NTCI. Fifth, the prominent role of CTLA4 in immunosuppressive stage of sepsis and its reversal by the PGT with NTCI peptide. Sixth, the control of the Complement Receptor 2 by the NTCI peptide, which extends its potential utility to sepsis caused by the Dengue Virus and similar hemorrhagic viruses.

The estimated 1 million of the immunocompromised patients with asplenia or hyposplenia in the USA, who are particularly vulnerable to sepsis (5), remain of great concern. Therefore, the positive impact of the PGT with NTCI peptide on the antibacterial immunity of the spleen, lungs, and blood in the preclinical model of sepsis is highly meritorious. The lungs constitute the initial site of pneumococcal infections in the spleen-immunocompromised patients. Therefore, 11-fold enhancement of the antibacterial immunity in the lungs by the PGT with NTCI peptide (4) may provide another genomic shield against bacterial infections of the lungs in these patients.

**Figure 5:**
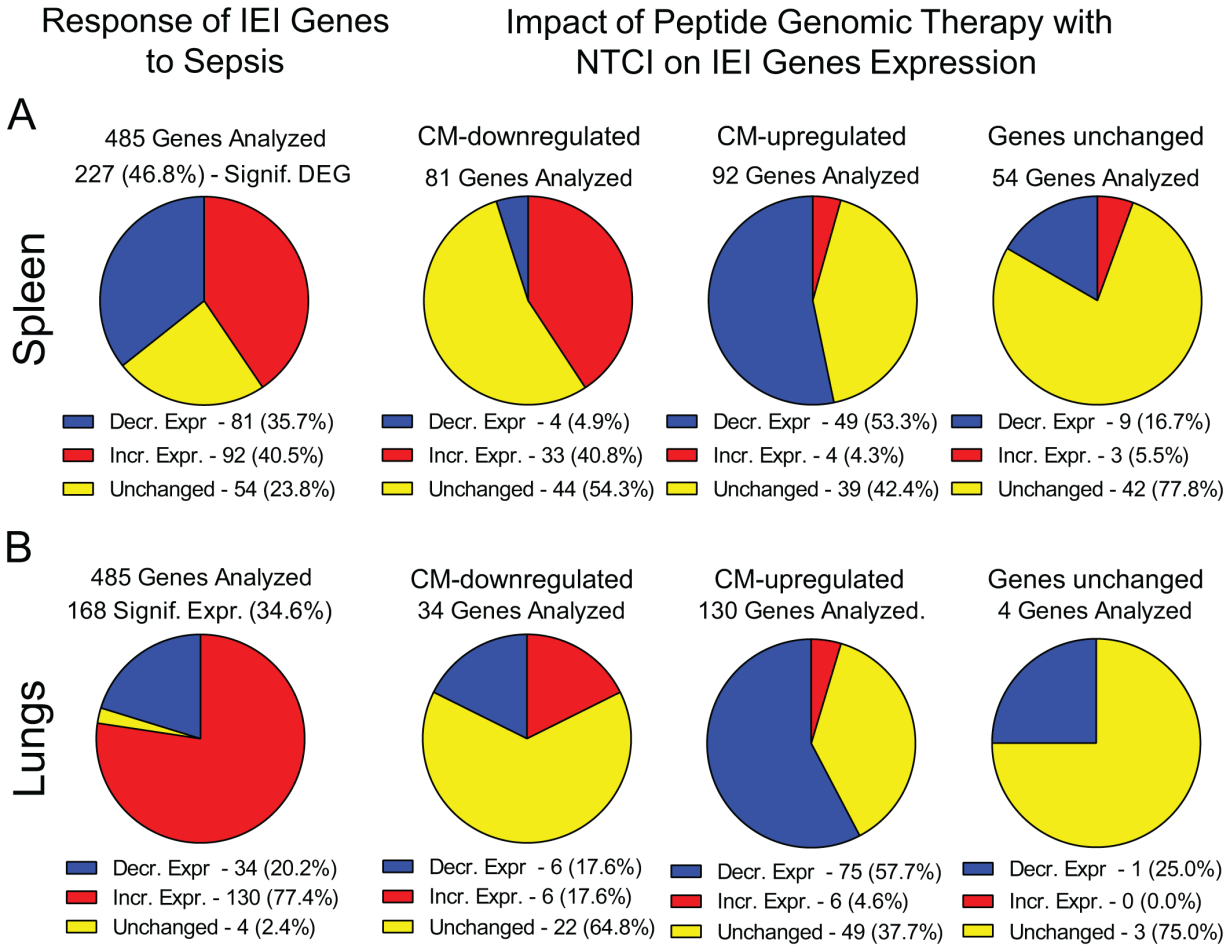
Response of IEI genes to polymicrobial sepsis in the spline and lungs. Blue – genes with decreased expression, red – genes with increased expression, yellow – genes with unchanged expression. Please note that the pie charts are constructed based on genes significantly expressed (padj < 0.05, see Materials and Methods for details). **(A)** The response of IEI genes in spleen to sepsis and the impact of Peptide Genomic Therapy with NTCI peptide. Mice were CM infected and treated with saline or meropenem with NTCI according to “Survival Protocol”. Mice were euthanized 26 hrs. after CM infection (see **Figure 1** and Materials and Methods for details). **(B)** The response of IEI genes in lungs to sepsis and the impact of Peptide Genomic Therapy with NTCI peptide. Mice were CM infected and treated with saline or NTCI according to the short treatment protocol for “Lungs NGS Analysis”. Mice were euthanized 6 hrs. after CM infection (see **Figure 1** and Materials and Methods for details).

We found that 173 IEI genes in the spleen and 164 IEI genes in the lungs responded to sepsis. Among them, expression of 36% genes was decreased whereas expression of 40.5% genes was increased in the spleen (**Figure 5A**). In the lungs, 20% of IEI genes displayed decreased expression and 77% had increased expression in sepsis (**Figure 5B**). Importantly, the PGT with NTCI peptide reprogrammed 102 IEI genes in the spleen and 94 IEI genes in the lungs (**Figure 4**). It is of relevance to the effective management of sepsis that the NTCI peptide suppressed the mediators of inflammation in the blood (IL-6, IL-10, TNFα, Interferon γ, and MCP1). Cumulatively, the Peptide Genomic Therapy with NTCI peptide, added to the antibiotic therapy, counteracts the polymicrobial sepsis in this preclinical model.

We were surprised to find that the *Il10* and *Nlrp12*, respectively, top the roster of the spleen’s IEI genes downregulated and upregulated by the NTCI peptide (see **Supplementary Tables S1, S2**). *Il10* gene expression depends on the panoply of transcription factors, including specificity protein (Sp), signal transducers and activators of transcription (STATs), interferon regulatory factors (IRFs), activator protein (AP), cAMP response element binding protein (CREB), CCATT enhancer/binding protein (C/EBP), c-musculoaponeurotic fibrosarcoma factor (c-MAF), and nuclear factor κB (NF-κB). These transcription factors are considered essential or critical in the *Il10* regulation (41). The NTCI peptide treatment also impeded at least 5 of these transcription factors on their path to the genome (16). Consequently, the PGT with NTCI peptide reduced the plasma level of IL-10 in septic animals to the baseline. We also extended the “nuclear blockade” by PGT with NTCI peptide to the gene encoding CTLA4, the main immunosuppressive checkpoint. It was induced 21 times in polymicrobial sepsis (see above), thereby contributing to the lowering of anti-bacterial immunity. The NTCI peptide-induced downregulation of the *Ctla4* gene prevented its immunosuppressive action in polymicrobial sepsis.

The decreased CR2 levels were reported in Dengue Virus Fever patients and correlated with the lowering of blood platelet counts. The 4-fold upregulation of the CR2 gene in our study, as well as the NTCI peptide-linked correction of thrombocytopenia in sepsis, as previously reported by us (15), bode well for the NTCI peptide’s utility in sepsis caused not only by bacteria but also by Dengue Virus, and other hemorrhagic viral infections.

In summary, we report the unprecedented induction of the human Inborn Errors of Immunity gene orthologs in the spleen and lungs in sepsis. Spleen, a major blood-filtering immune organ, plays a key role in the susceptibility and response to the invasive bacterial infections evolving into sepsis. Patients with asplenia and hyposplenia face the burdensome risk of sepsis. As the lungs are the site of viral and bacterial pneumonias leading to sepsis in asplenic and hyposplenic patients, the enhancement of the anti-bacterial immunity in both organs (and in blood) by the Peptide Genomic Therapy with NTCI peptide provides a potentially effective adjunct to the anti-microbial treatment. We also found that the PGT with NTCI peptide controls the genes that were not previously highlighted as exceptionally strong responders to sepsis. The significant reprogramming of these genes by the NTCI peptide contributes to the increased bacterial clearance thereby almost doubling the survival in this preclinical model of sepsis. Thus, our findings of the response of human Inborn Errors of Immunity gene orthologs to experimental sepsis in the two major organs, spleen and lungs, and their extraordinary control by the Peptide Genomic Therapy with NTCI peptide, present a new approach to the intractable problem of sepsis in the immunocompromised as well as immunocompetent patients.

## Materials and methods

### Synthesis and purification of the cell-penetrating nuclear transport checkpoint inhibitor

The cell-penetrating NTCI peptide, cSN50.1 (AAVALLPAVLLALLAPCVQRKRQKLMPC, 2986 Da) was synthesized as described elsewhere (42). The peptide chain was assembled through Solid Phase Peptide Synthesis (SPPS) according to standard Fmoc chemistry protocols using an automated peptide synthesizer FOCUS XC (AAPPTec, Louisville, KY). Crude peptides were removed from the resin with a TFA cleavage cocktail and purified by dialysis against double-distilled water in 1 kDa membrane (Spectra/Por 7; Spectrum Laboratories, Rancho Dominguez, CA). The purity and structure of the final products were verified respectively by an analytical C_18_ RP HPLC (Beckman Coulter GOLD System, Brea, CA) and MALDI mass spectroscopy (Voyager Elite; PerSeptive Biosystems, Framingham, MA). Before treatment, the NTCI peptide (cSN50.1) was solubilized in sterile water (half the final volume) and diluted with sterile saline to a final concentration of 3.3 mg/ml.

### Preparation of cecal microbiome stock

The preparation and titration of CM stock was previously described in (4). Briefly, 8 – 10-week-old C57BL/6 mice (Jackson Laboratories, Bar Harbor, ME) were euthanized by over inhalation of isoflurane followed by cervical dislocation. The cecum was removed; the gut microbiome content was extruded into a separate pre-weighed vial and processed as described in (4). Before each intraperitoneal injection, several vials were combined to suffice injection of 1.5 x 10^6^ CFUs/kg. In parallel, a sample of 20 µl from pooled CM stock was tested for quality control (4).

### Animal studies

As previously reported (4), the animal experiments were carried out in compliance with the ARRIVE guidelines and in strict conformity with the Guide for the Care and Use of Laboratory Animals of the National Institutes of Health. The submitted protocols were approved by the Vanderbilt University Institutional Animal Care and Use Committee. In all animal assays, an adapted model of nonsurgical polymicrobial peritonitis was used (15, 22). During the experiments, mice were closely monitored and euthanized by isoflurane over inhalation followed by cervical dislocation upon expression of the signs of moribund state. Survivors were euthanized at the experimental end point (168h). The experimental groups were selected using a double blinded randomization method (18). Each experiment was performed at least twice to assure statistical significance and experimental reproducibility. Blood was collected from the saphenous vein.

### Determination of LD_60_ for cecal microbiome

As previously reported (4), randomly grouped (n=6) 8-10-week-old female C57Bl/6J mice (The Jackson Laboratory, Bar Harbor, ME) were infected by single intraperitoneal (i.p.) injection of a CM stock corresponding to 1.1×10^6^, 1.25×10^6^, 1.45×10^6^, 1.6×10^6^, or 1.75×10^6^ CFU/kg and were treated with meropenem (25 mg/kg, subcutaneously (s.c.) every 12h). Animals were closely monitored throughout the experiment and euthanized upon expression of signs of moribund state. Survivors were euthanized at 168 h post CM challenge.

### Infection and treatment

The experimental groups: sham-challenged (Sham), CM-infected and treated with saline (CM); CM-infected and treated with meropenem and saline (CM+Mero); and CM-infected and treated with meropenem and NTCI (CM+Mero+NTCI), were comprised of randomly assigned 8 – 10-week-old female C57Bl/6J mice (The Jackson Laboratory, Bar Harbor, ME) (18). Mice were challenged with 5% dextrose (Sham) or infected by a single (i.p.) injection of a CM stock of 1.5x10^6^ CFU/kg corresponding to the LD_60_ dose. Antibiotic therapy with meropenem (25 mg/kg administered s.c.) began 1h post infection (CM challenge) and was continued every 12 hrs. until euthanasia. Treatment with i.p. injection of NTCI peptide [cSN50.1, 33 μg/g/injection in 200 μl 0.45% NaCl (15)] or vehicle (0.45% NaCl, 200 μl) begun 1h after infection and was continued every 3 hrs. for the first 24h, every 6 hrs. during the 2-nd day of experiment, then every 12 hrs. until euthanasia at 168h post infection.

For the NGS analysis of the spleen and determination of bacterial dissemination in the spleen, mice were euthanized at 26 hrs. post CM-infection. For the NGS analysis of the lungs, mice were treated with the i.p. injection of the NTCI peptide (cSN50.1), 33 μg/g/injection in 200 μl 0.45% NaCl or 0.45% NaCl (200 μl) as vehicle control, at 30 min before and 30 min, 1.5h, 2.5h, 3.5h, and 5h after CM challenge. Mice were euthanized 6 hrs. after CM-infection (**Figure 1**).

### Body temperature measurement

Mice body core temperature was measured at Vanderbilt University Mouse Metabolic Phenotyping Center. Sterile, battery free, RFID temperature transponder (IPTT-300) was implanted under the dorsal skin according to the manufacturer’s instruction. Animals were infected and treated as described above. Temperature was recorded by a reader placed directly under the cage before (0h) and after the infection at 1h, 3h, 6h, 9h, 12h, 15h, 18h, 24h, 30h, 36h, 42h, 48h, 60h,72h, 96, 120h, 144h, and 168h.

### Next-generation sequencing

We followed the protocol as previously reported (4). Briefly, mice were euthanized at 6 hrs. (for lungs) or 26 hrs. (for spleen) post CM-challenge and organs were processed for total RNA extraction as described therein. Three samples from each experimental condition (Sham + Saline, CM + Saline, CM + cSN50.1) from lungs and spleen were submitted to the Vanderbilt Technologies for Advanced Genomics (VANTAGE) Core for RNA sequencing analysis. RNA integrity numbers (RIN) were measured using TapeStation system (Agilent Technologies, Santa Clara, CA) and total RNA was processed into Stranded mRNA (NEB) Library. RNA sequencing was performed using Illumina NovaSeq6000 (San Diego, CA). The quality control of RNA preparation was analyzed by DRAGEN RNA Pipeline (v3.7.5). The count normalization with DESeq2 was used as a normalization method for gene expression analysis. The differential expression of the genes was analyzed using Illumina DRAGEN Secondary Analysis software. The expressions of approximately 15.5 thousand genes were analyzed in each organ and condition (**Supplementary Figure S3**). The numbers of genes considered as significantly expressed were determined by a false discovery rate (FDR) set to *p*_adj_<0.05. Genes with increased/decreased expression, as compared to the experimental control group (Sham), were selected from the pool of genes significantly expressed displaying values of log_2_(fold change) greater/less than center of distribution ± standard deviation determined by Gaussian distribution plot (**Supplementary Figure S2**). Genes with log_2_(FC) values located within the range established by standard deviation were considered as genes with unchanged expression (**Supplementary Figure S2**).

The analyzed conditions were as follows: CM-infected and treated with saline (CM) vs. Sham-challenged and treated with saline (Sham) to evaluate the impact of sepsis on gene expression, and CM-infected and treated with the NTCI peptide (cSN50.1) (CM+Mero+NTCI) vs. Sham-challenged and treated with saline (sham) to evaluate the impact of PGT with the NTCI on sepsis-induced gene expression.

### Determination of bacterial dissemination in the spleen

We followed the protocol as previously reported (4). Mice were euthanized 26 hrs. post CM infection (see Animal Studies for treatment details) and spleen was collected for the analysis of bacterial count. Isolated organs were externally sterilized by brief immersion in 70% ethanol, washed with cold sterile PBS, and placed in pre-weighed vials containing 0.5 ml cold sterile PBS. Tissue samples were weighed, and net weights were recorded. Organs were homogenized with a disposable sterile plastic homogenizer and suspension passed through the 70 µm cell strainers. 50 µl of suspension was serially diluted with sterile PBS resulting in the final concentration of 1:10,000. The diluted samples (200 µl) were applied to TSA + 5% sheep blood agar plate for overnight incubation at 37ºC. The colonies (non-viridans) were counted down and then converted into or CFU/g of a wet organ mass (spleen).

### Cytokines/chemokine assay

The proteins levels of cytokines TNF-α, IL-6, IL-10, and IFN-γ, and chemokine MCP-1 were measured in blood plasma before and 6 and 24 hrs. post CM infection [CM, CM+Meropenem (Mero), and CM+Mero+NTCI)]. A cytometric bead array (BD Biosciences) assay was performed and analyzed in the Vanderbilt University Medical Center Flow Cytometry Shared Resource, as previously described (15).

### Statistical analysis

Normal distribution of data sets was verified using normal probability plot (q-q) and Kolmogorov-Smirnov Normality Test. A statistical analysis was performed using Prism 6 software (GraphPad, Boston, MA). Plasma levels of cytokines were analyzed by ordinary one-way ANOVA with an uncorrected Fisher’s LSD test for a multiple comparison. Bacterial dissemination in spleen was analyzed by nonparametric t test with Mann–Whitney rank comparison. The data is presented as a mean ± SEM. *p* values of < 0.05 were considered significant. The standard deviation and the center of the log_2_(FC) values distribution in NGS analyzed data was determined by the Gaussian plot (**Supplementary Figure S3**). Log_2_(FC) values were rounded down to the nearest decimal point (0.1) and equal values were added up. These numbers were then plotted against log_2_(FC) and analyzed by a Gaussian/Lorentzian function using Prism 6 software (GraphPad, Boston, MA).

## Data availability statement

All data generated or analyzed during this study are included in this published article and its supplementary information files. The NGS Data is deposited in the NBCI Gene Expression Omnibus (GEO) repository of high throughput sequencing data. Accession numbers are GSE308045 (spleen) and GSE239388 (lungs).
