# Supplementary material for: The peptide genomic therapy increases antibacterial immunity and survival in sepsis by reprograming the gene orthologs of human immunodeficiencies in the spleen and lungs
Source: Front Immunol. 2025 Oct 28;16:1635081. doi: 10.3389/fimmu.2025.1635081 (PMC12602443; doi:10.3389/fimmu.2025.1635081)
Supplement: Supplementary file 1 [file DataSheet1.pdf]

Supplemental Materials

The Peptide Genomic Therapy Increases Antibacterial Immunity and Survival in Sepsis by Reprograming the Gene Orthologs of Human Immunodeficiencies in the Spleen and Lungs

Huan Qiao[1†], Jozef Zienkiewicz[1,2†], Yan Liu[1,2], and Jacek Hawiger[1,2,3,*]

[1] Vanderbilt University School of Medicine, Department of Medicine, Division of Allergy, Pulmonary and Critical Care Medicine, Nashville, Tennessee, United States of America

[2] Department of Veterans Affairs, Tennessee Valley Health Care System, Nashville, Tennessee, United States of America

[3] Vanderbilt University School of Medicine, Department of Molecular Physiology and Biophysics, Nashville, Tennessee, United States of America

[†] These authors contributed equally to this work and share first authorship.

* Correspondence and requests for materials should be addressed to:
Jacek Hawiger (ORCID: 0000-0003-2721-6859)
Vanderbilt University Medical Center
21st Avenue South, T-1218, MCN
Nashville, TN 37232, USA
phone: +1 (615) 828-8718
e-mail: jack.hawiger@vanderbilt.edu

Supplemental Figure SF1

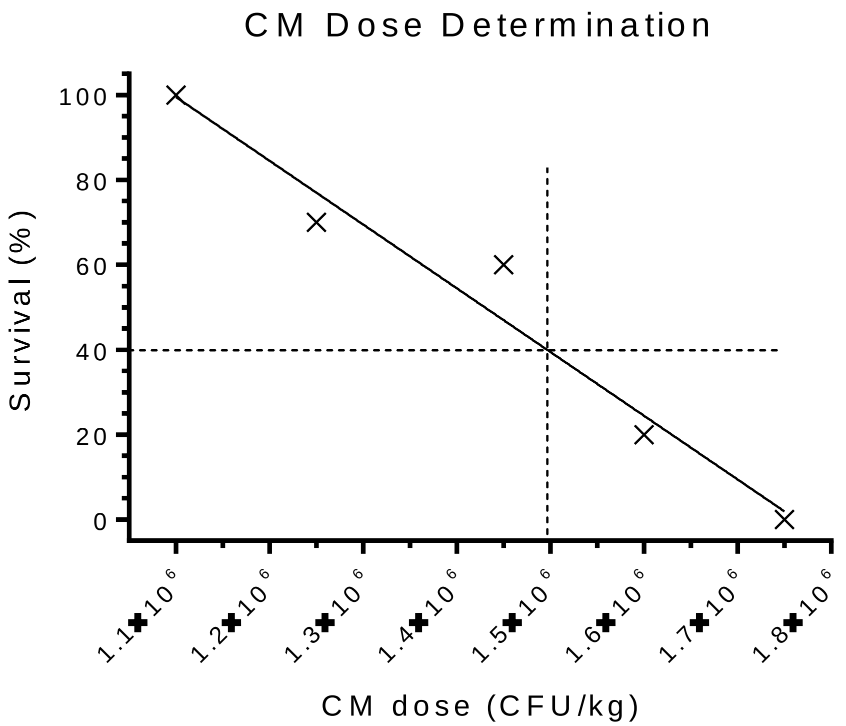

Suppl. Fig. SF1. Determination of Cecal Microbiome Lethal Dose Causing 60% Mortality. 8-week-old mice were challenged with a single i.p. injection of CM at a dose corresponding to 1.1×10^6, 1.25×10^6, 1.45×10^6, 1.6×10^6, or 1.75×10^6 CFU/kg and were treated with meropenem (25 mg/kg, s.c. every 12h). Mice were observed for 7 days after the CM-challenge and the survival was recorded. A dose- dependent survival represents a linear correlation according to the following equation: Survival = $-1.5\times10^4 \times$ CFU + 264.8 with R^2=0.9621 (p=0.027). The LD_{60} corresponds to 1.5×10^6 CFU/kg of body mass.

Supplemental Figure SF2

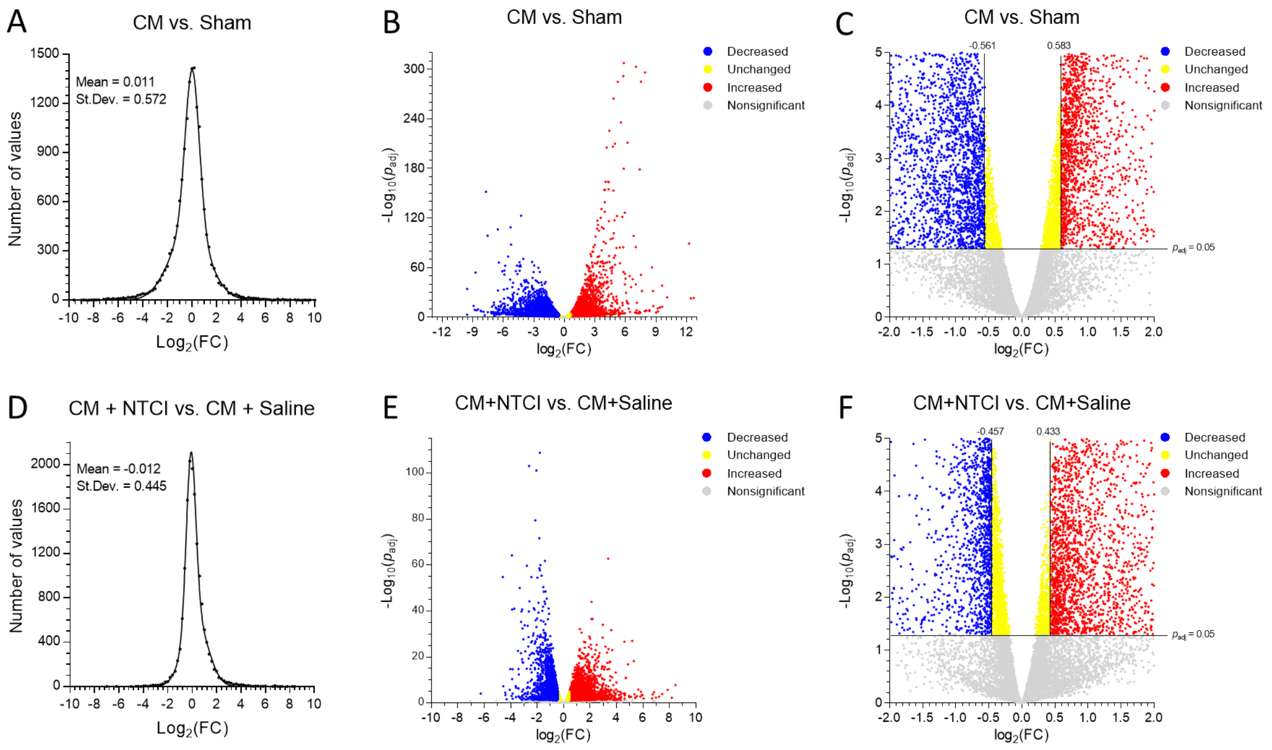

Suppl. Fig. S2. Distribution of the log2(FC) values in Gaussian and Volcano Plots. **A** and **D**. Gaussian distributions of log2(FC) values were used to determine the center of distribution and the standard deviation for selection of genes with increased (Red), decreased (Blue), and unchanged (Yellow) expression. The Log2(FC) values were rounded down to the nearest decimal point (0.1) and the equal values were added up. Data points were plotted against the log2(FC) (see Materials and Methods for details). **B** and **E**. Volcano plots represent distribution of log2(FC) values expressed as -$\log_{10}(p_{adj})$ and are used to visualize expression of upregulated or downregulated genes corelated with their statistical significance. **C** and **F**. Enlarged central portions of corresponding volcano plots demonstrating boundaries of genes with increased, decreased or unchanged expression limited by standard deviation determined by Gaussian plots. Genes expression with $p_{adj} > 0.05$ are considered statistically nonsignificant (Grey).

Supplemental Figure SF3

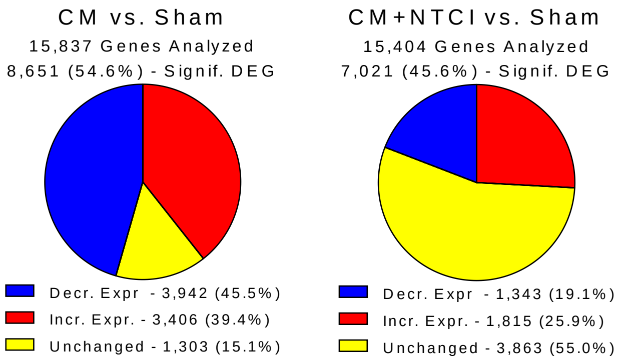

Suppl. Fig. S3. Genomic Response to Polymicrobial Sepsis in the Spleen. The response of the spleen genome to sepsis in untreated and the NTCI-treated animals indicates the NTCI-increased pool of unchanged genes from 15.1% to 55%, respectively. The overall view of the gene expression in the spleen of CM-infected mice following treatment with saline (CM vs Sham) or the Peptide Genomic Therapy with NTCI (CM+NTCI vs Sham). Blue – genes with decreased expression, Red – genes with increased expression, Yellow – genes with unchanged expression. Please note that the pie charts are constructed based on genes significantly expressed ($p_{adj} < 0.05$, see Materials and Methods for details).
