# Supplementary material for: The peptide genomic therapy increases antibacterial immunity and survival in sepsis by reprograming the gene orthologs of human immunodeficiencies in the spleen and lungs
Source: Front Immunol. 2025 Oct 28;16:1635081. doi: 10.3389/fimmu.2025.1635081 (PMC12602443; doi:10.3389/fimmu.2025.1635081)
Supplement: Supplementary file 2 [file DataSheet2.pdf]

Suppl. Tab. ST1A. IEI Genes with Inceased Expression in Spleen of CM-Infected Mice

			Lungs	
	Gene ID	Gene Name	Saline	NTCI
1	*Il10*	interleukin 10	170	26
2	*Sema3e*	sema domain, immunoglobulin domain (Ig), short basic domain, secreted, (semaphorin) 3E	34	74
3	*Il17f*	interleukin 17F	31	6.5
4	*Ctla4*	cytotoxic T-lymphocyte-associated protein 4	21	4.3
5	*Il1rn*	interleukin 1 receptor antagonist	21	8.9
6	*Tnfaip3*	tumor necrosis factor, alpha-induced protein 3	9.1	2.6
7	*Tnfrsf9*	tumor necrosis factor receptor superfamily, member 9	8.8	2.4
8	*Cd70*	CD70 antigen	8.3	4.7
9	*Irf7*	interferon regulatory factor 7	6.7	1.8
10	*Ifng*	interferon gamma	5.4	-2.0
11	*Usp18*	ubiquitin specific peptidase 18	5.2	1.8
12	*Cfd*	complement factor D	5.1	-1.2
13	*Nos2*	nanos C2HC-type zinc finger 2	5.1	2.8
14	*Socs1*	suppressor of cytokine signaling 1	5.1	2.1
15	*Il2ra*	interleukin 2 receptor, alpha chain	4.9	3.4
16	*Tet2*	tet methylcytosine dioxygenase 2	4.8	1.4
17	*Il12rb1*	interleukin 12 receptor, beta 1	4.7	1.7
18	*Nfkbia*	nuclear factor of kappa light polypeptide gene enhancer in B cells inhibitor, alpha	4.5	1.8
19	*Cfb*	complement factor B	4.3	2.3
20	*Isg15*	ISG15 ubiquitin-like modifier	3.8	1.1
21	*Tnfrsf4*	tumor necrosis factor receptor superfamily, member 4	3.8	2.2
22	*Hmox1*	heme oxygenase 1	3.4	1.4
23	*Stat3*	signal transducer and activator of transcription 3	3.3	1.9
24	*Tcirg1*	T cell, immune regulator 1, ATPase, H+ transporting, lysosomal V0 protein A3	3.2	2.0
25	*Tap1*	transporter 1, ATP-binding cassette, sub-family B (MDR/TAP)	3.1	1.1
26	*Ifih1*	interferon induced with helicase C domain 1	3.0	1.1
27	*C4a*	complement C4A (Rodgers blood group)	2.9	1.9
28	*Stat2*	signal transducer and activator of transcription 2	2.9	1.1
29	*Il21*	interleukin 21	2.8	1.9
30	*Ung*	uracil DNA glycosylase	2.8	1.4
31	*Atad3a*	ATPase family, AAA domain containing 3A	2.7	1.9
32	*Rel*	reticuloendotheliosis oncogene	2.7	1.1
33	*Samhd1*	SAM domain and HD domain, 1	2.6	-1.2
34	*Sp110*	Sp110 nuclear body protein	2.6	1.1
35	*Gfi1*	growth factor independent 1 transcription repressor	2.5	1.1
36	*Fas*	fatty acid synthase	2.4	1.4
37	*Hax1*	HCLS1 associated X-1	2.4	2.9
38	*Il23r*	interleukin 23 receptor	2.4	-1.9
39	*Nlrp3*	NLR family, pyrin domain containing 3	2.4	1.2
40	*Psenen*	presenilin enhancer gamma secretase subunit	2.4	2.4
41	*Tlr7*	toll-like receptor 7	2.4	-1.0
42	*Trex1*	three prime repair exonuclease 1	2.4	-1.0
43	*Chd7*	chromodomain helicase DNA binding protein 3	2.3	1.3
44	*Dnmt3b*	DNA methyltransferase 3B	2.3	1.4
45	*Cd40*	CD40 antigen	2.2	-1.2
46	*Fanca*	Fanconi anemia, complementation group A	2.2	-1.1
47	*Rag1*	recombination activating 1	2.2	1.1
48	*Rpsa*	ribosomal protein SA	2.2	1.7
49	*Snx10*	sorting nexin 10	2.2	1.6
50	*Tapbp*	TAP binding protein	2.2	-1.1
51	*Dbr1*	debranching RNA lariats 1	2.1	1.1
52	*Gata2*	GATA binding protein 2	2.1	2.0
53	*Il18bp*	interleukin 18 binding protein	2.1	2.4
54	*Cfh*	complement component factor h	2.0	2.5
55	*Csf2rb*	colony stimulating factor 2 receptor, beta, low-affinity (granulocyte-macrophage)	2.0	2.3
56	*Dkc1*	dyskeratosis congenita 1, dyskerin	2.0	1.3
57	*Irf4*	interferon regulatory factor 4	2.0	1.5
58	*Mad2l2*	MAD2 mitotic arrest deficient-like 2	2.0	1.5
59	*Nhp2*	NHP2 ribonucleoprotein	2.0	1.3
60	*Tbk1*	TANK-binding kinase 1	2.0	1.1
61	*Tnfrsf11a*	tumor necrosis factor receptor superfamily, member 11a, NFKB activator	2.0	1.5
62	*Trnt1*	tRNA nucleotidyl transferase, CCA-adding, 1	2.0	1.3
63	*Dclre1c*	DNA cross-link repair 1C	1.9	1.2

64	*Myd88*	myeloid differentiation primary response gene 88	1.9	1.5
65	*Pold2*	polymerase (DNA directed), delta 2, regulatory subunit	1.9	1.3
66	*Atm*	ataxia telangiectasia mutated	1.8	1.5
67	*Dnajc21*	DnaJ heat shock protein family (Hsp40) member C21	1.8	1.3
68	*Ercc6l2*	excision repair cross-complementing rodent repair deficiency, complementation group 6 like 2	1.8	1.7
69	*Fance*	Fanconi anemia, complementation group E	1.8	1.2
70	*Il17rc*	interleukin 17 receptor C	1.8	2.8
71	*Irf2bp2*	interferon regulatory factor 2 binding protein 2	1.8	1.0
72	*Polr3a*	polymerase (RNA) III (DNA directed) polypeptide A	1.8	1.2
73	*Serping1*	serine (or cysteine) peptidase inhibitor, clade G, member 1	1.8	1.7
74	*Tada2a*	transcriptional adaptor 2A	1.8	1.6
75	*Cdca7*	cell division cycle associated 7	1.7	1.5
76	*Foxp3*	forkhead box P3	1.7	1.6
77	*Hells*	helicase, lymphoid specific	1.7	2.3
78	*Mysm1*	myb-like, SWIRM and MPN domains 1	1.7	1.2
79	*Nop10*	NOP10 ribonucleoprotein	1.7	1.2
80	*Tnfrsf1a*	tumor necrosis factor receptor superfamily, member 1a	1.7	1.8
81	*Usb1*	U6 snRNA biogenesis 1	1.7	1.3
82	*C2*	proteasome subunit alpha 1	1.6	1.3
83	*Cd46*	CD46 antigen, complement regulatory protein	1.6	-1.1
84	*Icos*	inducible T cell co-stimulator	1.6	1.0
85	*Mapk8*	mitogen-activated protein kinase 8	1.6	1.0
86	*Nfe2l2*	nuclear factor, erythroid derived 2, like 2	1.6	1.2
87	*Rtel1*	regulator of telomere elongation helicase 1	1.6	1.1
88	*Sppl2a*	signal peptide peptidase like 2A	1.6	-1.1
89	*Tirap*	toll-interleukin 1 receptor (TIR) domain-containing adaptor protein	1.6	1.5
90	*Ttc37*	SKI3 subunit of superkiller complex	1.6	1.8
91	*Acp5*	acid phosphatase 5, tartrate resistant	1.5	1.5
92	*Ak2*	adenylate kinase 2	1.5	1.2
93	*Bach2*	BTB and CNC homology, basic leucine zipper transcription factor 2	1.5	-1.2
94	*Ctsc*	cathepsin C	1.5	1.3
95	*Cxcr4*	C-X-C motif chemokine receptor 4	1.5	1.2
96	*Ino80*	INO80 complex subunit	1.5	1.2
97	*Malt1*	MALT1 paracaspase	1.5	-1.2
98	*Map3k14*	mitogen-activated protein kinase kinase kinase 14	1.5	-1.1
99	*Parn*	poly(A)-specific ribonuclease (deadenylation nuclease)	1.5	1.1
100	*Rbck1*	RanBP-type and C3HC4-type zinc finger containing 1	1.5	-1.1

Suppl. Tab. ST1B. IEI Genes with Decreased Expression in Spleen of CM-Infected Mice

			Lungs	
	Gene ID	Gene Name	Saline	NTCI
1	*Nlrp12*	NLR family, pyrin domain containing 12	-40	-11
2	*Cxcr2*	C-X-C motif chemokine receptor 2	-35	-4.1
3	*Cr2*	defensin, alpha, 2	-12	-3.1
4	*C6*	complement component 6	-6.0	-1.9
5	*Ms4a1*	membrane-spanning 4-domains, subfamily A, member 2	-5.8	-2.3
6	*C8g*	complement component 8, gamma polypeptide	-5.6	-1.3
7	*Ciita*	class II transactivator	-5.5	-3.0
8	*Havcr2*	hepatitis A virus cellular receptor 2	-5.0	-4.1
9	*Tnfsf13*	tumor necrosis factor (ligand) superfamily, member 13	-4.8	1.1
10	*Cd3g*	CD3 antigen, gamma polypeptide	-4.6	-2.2
11	*Cd8a*	CD8 subunit alpha	-4.3	-2.0
12	*Cd40lg*	CD40 ligand	-4.1	-2.1
13	*Tmc8*	transmembrane channel-like gene family 8	-3.8	-1.8
14	*Tfrc*	transferrin receptor	-3.6	-3.0
15	*Aicda*	activation-induced cytidine deaminase	-3.4	-2.1
16	*Cd27*	CD27 antigen	-3.4	-1.5
17	*Rad51c*	RAD51 paralog C	-3.3	-1.3
18	*Rfxank*	regulatory factor X-associated ankyrin-containing protein	-3.3	-1.3
19	*Tert*	telomerase reverse transcriptase	-3.3	1.1
20	*Cd3e*	CD3 antigen, epsilon polypeptide	-3.0	-1.6
21	*Cd79b*	CD79B antigen	-3.0	-2.4
22	*Card11*	caspase recruitment domain family, member 11	-2.8	-2.6
23	*Cd3d*	CD3 antigen, delta polypeptide	-2.8	-1.9
24	*Csf3r*	colony stimulating factor 3 receptor	-2.8	-2.0
25	*Cd79a*	CD79A antigen (immunoglobulin-associated alpha)	-2.7	-2.0
26	*Slc46a1*	solute carrier family 46, member 1	-2.7	-1.0
27	*Elane*	elastase, neutrophil expressed	-2.6	-3.1
28	*Ncf1*	neutrophil cytosolic factor 1	-2.6	-1.5
29	*Rasgrp1*	RAS guanyl releasing protein 1	-2.6	-1.6
30	*Irak4*	interleukin-1 receptor-associated kinase 4	-2.5	1.1
31	*Prf1*	perforin 1 (pore forming protein)	-2.5	-2.3
32	*Sh3kbp1*	SH3-domain kinase binding protein 1	-2.5	-1.4
33	*Cfhr2*	complement factor H-related 2	-2.4	-2.3
34	*Igkc*	immunoglobulin kappa constant	-2.4	-1.8
35	*Sh2d1a*	SH2 domain containing 1A	-2.3	-1.8
36	*Lck*	lymphocyte protein tyrosine kinase	-2.2	-1.6
37	*Rnaseh2c*	ribonuclease H2, subunit C	-2.2	-1.4
38	*Ube2t*	ubiquitin-conjugating enzyme E2T	-2.2	-1.3
39	*Unc13d*	unc-13 homolog D	-2.2	-1.5
40	*Bcl11b*	B cell leukemia/lymphoma 11B	-2.1	-1.5
41	*Cd247*	CD247 antigen	-2.1	-1.6
42	*Fadd*	Fas associated via death domain	-2.1	-1.1
43	*Plcg2*	phospholipase C, gamma 2	-2.1	-1.4
44	*Tyk2*	tyrosine kinase 2	-2.1	-1.4
45	*Coro1a*	coronin, actin binding protein 1A	-2.0	-1.5
46	*Dock2*	dedicator of cyto-kinesis 2	-2.0	-1.6
47	*Ikzf3*	IKAROS family zinc finger 3	-2.0	-1.8
48	*Lyst*	lysosomal trafficking regulator	-2.0	-1.2
49	*Cd55*	CD55 molecule, decay accelerating factor for complement	-1.9	-1.5
50	*Fancc*	Fanconi anemia, complementation group C	-1.9	-1.3
51	*Lamtor2*	late endosomal/lysosomal adaptor, MAPK and MTOR activator 2	-1.9	-1.1
52	*Magt1*	magnesium transporter 1	-1.9	-1.0
53	*Nhej1*	non-homologous end joining factor 1	-1.9	-1.1
54	*Pole2*	polymerase (DNA directed), epsilon 2 (p59 subunit)	-1.9	-1.6
55	*Zap70*	zeta-chain (TCR) associated protein kinase	-1.9	-1.5
56	*Cd19*	CD19 antigen	-1.8	-1.5
57	*Man2b2*	mannosidase 2, alpha B2	-1.8	-1.2
58	*Pola1*	polymerase (DNA directed), alpha 1	-1.8	-1.4
59	*Atp6ap1*	ATPase, H+ transporting, lysosomal accessory protein 1	-1.7	-1.3
60	*Cfi*	complement component factor i	-1.7	1.0
61	*Cib1*	calcium and integrin binding 1	-1.7	-1.2
62	*Cybc1*	cytochrome b 245 chaperone 1	-1.7	-1.2
63	*Dock8*	dedicator of cytokinesis 8	-1.7	-1.3

64	*Ptprc*	protein tyrosine phosphatase receptor type C	-1.7	-1.5
65	*Slc7a7*	solute carrier family 7 (cationic amino acid transporter, y+ system), member 7	-1.7	1.2
66	*Tmc6*	transmembrane channel-like gene family 6	-1.7	-1.5
67	*Trac*	ATP binding cassette subfamily G member 5	-1.7	-1.6
68	*Card9*	caspase recruitment domain family, member 9	-1.6	-1.6
69	*Dnase1l3*	deoxyribonuclease 1-like 3	-1.6	-2.5
70	*Fancl*	Fanconi anemia, complementation group L	-1.6	-1.5
71	*Ikzf2*	IKAROS family zinc finger 2	-1.6	-1.2
72	*Il10rb*	interleukin 10 receptor, beta	-1.6	-1.1
73	*Il12b*	interleukin 12b	-1.6	-2.3
74	*Il2rg*	interleukin 2 receptor, gamma chain	-1.6	-1.5
75	*Ncf4*	neutrophil cytosolic factor 4	-1.6	-1.1
76	*Slc35c1*	solute carrier family 35, member C1	-1.6	-1.1
77	*Was*	Wiskott-Aldrich syndrome	-1.6	-1.3
78	*Ap3d1*	adaptor-related protein complex 3, delta 1 subunit	-1.5	-1.1
79	*Brca1*	breast cancer 1, early onset	-1.5	-1.9
80	*Cd28*	CD28 antigen	-1.5	-2.0
81	*Cd81*	CD81 antigen	-1.5	-1.0
82	*Cebpe*	CCAAT/enhancer binding protein epsilon	-1.5	1.7
83	*Elf4*	E74 like ETS transcription factor 4	-1.5	-1.3
84	*Extl3*	exostosin-like glycosyltransferase 3	-1.5	-1.3
85	*Fancd2*	Fanconi anemia, complementation group D2	-1.5	-1.4
86	*Fancg*	Fanconi anemia, complementation group G	-1.5	1.0
87	*Fanci*	Fanconi anemia, complementation group I	-1.5	-1.4
88	*Ighm*	immunoglobulin heavy constant mu	-1.5	-1.4
89	*Lig1*	ligase I, DNA, ATP-dependent	-1.5	-1.6
90	*Lig4*	ligase IV, DNA, ATP-dependent	-1.5	-1.0
91	*Nlrc4*	NLR family, CARD domain containing 4	-1.5	-1.0
92	*Prkdc*	protein kinase, DNA activated, catalytic polypeptide	-1.5	1.1
93	*Rac2*	Rac family small GTPase 2	-1.5	-1.1
94	*Rfwd3*	ring finger and WD repeat domain 3	-1.5	-1.3
95	*Rmrp*	RNA component of mitochondrial RNAase P	-1.5	-3.7
96	*Tcn2*	transcobalamin 2	-1.5	1.1
97	*Tlr8*	toll-like receptor 8	-1.5	1.2
98	*Tmem173*	stimulator of interferon response cGAMP interactor 1	-1.5	-1.2
99	*Adam17*	a disintegrin and metallopeptidase domain 17	-1.4	-1.0
100	*Atg4a*	autophagy related 4A, cysteine peptidase	-1.4	-1.4

Suppl. Tab. ST2A. Genes with Inceased Expression in Spleen of CM-Infected Mice

			Lungs	
	Gene ID	Gene Name	Saline	NTCI
1	*Exosc6*	exosome component 6	6,651	386
2	*Rsc1a1*	regulatory solute carrier protein, family 1	5,470	3,420
3	*Csf3*	colony stimulating factor 3 (granulocyte)	4,854	710
4	*Cxcl1*	C-X-C motif chemokine ligand 1	1,094	185
5	*Ptx3*	pentraxin related gene	772	233
6	*Psg22*	pregnancy-specific beta-1-glycoprotein 22	772	101
7	*Cxcl2*	C-X-C motif chemokine ligand 2	660	80
8	*Il22*	interleukin 22	584	25
9	*Gm6093*	predicted gene 6093	582	642
10	*Cxcl3*	C-X-C motif chemokine ligand 3	515	218
11	*Npy*	neuropeptide Y	424	26
12	*Gm14236*	predicted gene 14236	415	233
13	*Il6*	interleukin 6	387	74
14	*Igfbp1*	insulin-like growth factor binding protein 1	328	15
15	*Sprr2d*	small proline-rich protein 2D	325	17,306
16	*Pet117*	PET117 homolog	323	64
17	*Il17a*	interleukin 17A	267	16
18	*Pou4f2*	POU domain, class 4, transcription factor 2	264	180
19	*Rps27*	ribosomal protein S27	242	176
20	*Unc93a2*	unc-93 homolog A2	238	157
21	*Has1*	hyaluronan synthase 1	196	136
22	*Rps29*	ribosomal protein S29	184	147
23	*Ccl2*	C-C motif chemokine ligand 2	183	36
24	*Sptssb*	serine palmitoyltransferase, small subunit B	175	2,663
25	*Il10*	interleukin 10	170	26
26	*Slc6a14*	solute carrier family 6 (neurotransmitter transporter), member 14	164	282
27	*Gm2174*	predicted gene 2174	164	89
28	*Calcr*	calcitonin receptor	159	22
29	*AA467197*	expressed sequence AA467197	140	5.8
30	*Slco1c1*	solute carrier organic anion transporter family, member 1c1	137	21
31	*Ccl7*	C-C motif chemokine ligand 7	135	36
32	*Fgf23*	fibroblast growth factor 23	135	216
33	*Rps12*	ribosomal protein S12	130	100
34	*Tmem252*	transmembrane protein 252	130	21
35	*Plet1*	placenta expressed transcript 1	129	78
36	*Il19*	interleukin 19	121	28
37	*Ccl12*	C-C motif chemokine ligand 12	111	37
38	*Cxcl5*	C-X-C motif chemokine ligand 5	105	62
39	*Fosl1*	fos-like antigen 1	94	66
40	*Gm3417*	dynein light chain Tctex-type 2A3	87	55
41	*Mmp3*	matrix metallopeptidase 3	85	21
42	*Gjb3*	gap junction protein, beta 3	79	266
43	*Ereg*	epiregulin	77	255
44	*Lox*	lysyl oxidase	74	23
45	*Hmga2*	high mobility group AT-hook 2	72	195
46	*Crabp2*	cellular retinoic acid binding protein II	69	137
47	*Serpina3m*	serine (or cysteine) peptidase inhibitor, clade A, member 3M	67	79
48	*1700011B04Rik*	RIKEN cDNA 1700011B04 gene	64	10
49	*A730049H05Rik*	RIKEN cDNA A730049H05 gene	62	55
50	*Lyve1*	lymphatic vessel endothelial hyaluronan receptor 1	61	6.8
51	*Gjb4*	gap junction protein, beta 4	61	463
52	*Ccl3*	C-C motif chemokine ligand 3	61	18
53	*Rpl31*	ribosomal protein L31	57	47
54	*Pi15*	peptidase inhibitor 15	57	29
55	*Tnc*	tenascin C	56	21
56	*Phox2a*	paired-like homeobox 2a	55	37
57	*Gm9780*	Plac9, placenta specific 9	55	53
58	*Rpl24*	ribosomal protein L24	55	38
59	*Oacyl*	O-acyltransferase like	53	8.2
60	*Car4*	carbonic anhydrase 4	52	32
61	*Pla1a*	phospholipase A1 member A	51	28
62	*Ptgs2*	prostaglandin-endoperoxide synthase 2	51	28
63	*Slc26a9*	solute carrier family 26, member 9	49	100

64	*Rpl12*	ribosomal protein L12	47	33
65	*Gdf2*	growth differentiation factor 2	45	25
66	*Adamts4*	ADAM metallopeptidase with thrombospondin type 1 motif 4	44	33
67	*Rasd1*	RAS, dexamethasone-induced 1	43	2.9
68	*Lcn2*	lipocalin 2	43	27
69	*Csta2*	cystatin A family member 2	43	92
70	*Il11ra2*	interleukin 11 receptor subunit alpha 2	42	18
71	*Serpinb5*	serine (or cysteine) peptidase inhibitor, clade B, member 5	41	1,086
72	*Gm2007*	predicted gene 2007	40	13
73	*Npc1l1*	NPC1 like intracellular cholesterol transporter 1	40	-2.4
74	*Tex45*	stabilizer of axonemal microtubules 5	40	-1.9
75	*Dnmt3l*	DNA methyltransferase 3-like	39	26
76	*Serpina3c*	serine (or cysteine) peptidase inhibitor, clade A, member 3C	38	26
77	*Serpine1*	serine (or cysteine) peptidase inhibitor, clade E, member 1	38	34
78	*Rpl9*	ribosomal protein L9	38	29
79	*Cyp27b1*	cytochrome P450, family 27, subfamily b, polypeptide 1	37	36
80	*Erich2*	glutamate rich 2	36	19
81	*Gm10309*	predicted gene 10309	36	34
82	*Plac9a*	placenta specific 9	36	29
83	*1200007C13Rik*	RIKEN cDNA 1200007C13 gene	36	13
84	*Mmp13*	matrix metallopeptidase 13	35	3.2
85	*Adamts15*	ADAM metallopeptidase with thrombospondin type 1 motif 15	35	38
86	*Sema3e*	sema domain, immunoglobulin domain (Ig), short basic domain, secreted, (semaphorin) 3E	34	74
87	*Acod1*	aconitate decarboxylase 1	33	3.3
88	*Sele*	selectin, endothelial cell	32	23
89	*Tnip3*	TNFAIP3 interacting protein 3	32	10.0
90	*Ifitm5*	interferon induced transmembrane protein 5	32	13
91	*Gm13889*	predicted gene 13889	31	14
92	*Olfr1393*	olfactory receptor family 2 subfamily Y member 1G	31	2.2
93	*Lilr4b*	leukocyte immunoglobulin-like receptor, subfamily B, member 4B	31	9.3
94	*Unc93a*	unc-93 homolog A	31	13
95	*Rpl38*	ribosomal protein L38	31	22
96	*Gm29686*	predicted gene, 29686	31	15
97	*Il17f*	interleukin 17F	30	6.5
98	*Tmem171*	transmembrane protein 171	30	3.5
99	*Edn1*	endothelin 1	30	4.4
100	*Ccl4*	C-C motif chemokine ligand 4	29	9.2

Suppl. Tab. ST2B. Genes with Decreased Expression in Spleen of CM-Infected Mice

			Lungs	
	Gene ID	Gene Name	Saline	NTCI
1	*Clec4g*	C-type lectin domain family 4, member g	-750	-15
2	*Cckar*	cholecystokinin A receptor	-749	-13
3	*Redrum*	Redrum, erythroid developmental long intergenic non-protein coding transcript	-483	-51
4	*Gm14198*	predicted gene 14198	-451	-48
5	*Xcr1*	chemokine (C motif) receptor 1	-421	-71
6	*Rnf212*	ring finger protein 212	-401	-40
7	*Chrna6*	cholinergic receptor, nicotinic, alpha polypeptide 6	-359	-107
8	*Slc30a10*	solute carrier family 30, member 10	-298	-161
9	*Aknad1*	AKNA domain containing 1	-290	-5.6
10	*9830132P13Rik*	RIKEN cDNA 9830132P13 gene	-244	-11
11	*Gm37915*	predicted gene, 37915	-243	-12
12	*Hamp2*	hepcidin antimicrobial peptide 2	-229	-7.4
13	*Ccr3*	C-C motif chemokine receptor 3	-210	-5.8
14	*Fcer2a*	Fc receptor, IgE, low affinity II, alpha polypeptide	-187	-15
15	*Mobp*	myelin-associated oligodendrocytic basic protein	-184	-68
16	*4930572G02Rik*	RIKEN cDNA 4930572G02 gene	-183	-16
17	*Gm3336*	predicted gene 3336	-147	-11
18	*Gm31452*	predicted gene 31452	-144	-6.0
19	*Nme9*	RIKEN cDNA 1600029I14 gene	-139	-14
20	*1810053B23Rik*	RIKEN cDNA 1810053B23 gene	-139	-7.1
21	*Fn3k*	fructosamine 3 kinase	-138	-19
22	*Csl*	citrate synthase like	-130	-49
23	*Gm5096*	betaine--homocysteine S-methyltransferase 1B	-128	-6.9
24	*Cpne4*	copine IV	-127	-24
25	*Inmt*	indolethylamine N-methyltransferase	-125	-7.4
26	*Gm47252*	predicted gene, 47252	-125	-11
27	*Skint3*	selection and upkeep of intraepithelial T cells 3	-122	-1.1
28	*Crisp3*	cysteine-rich secretory protein 3	-122	-19
29	*Grin2b*	glutamate receptor, ionotropic, NMDA2B (epsilon 2)	-117	-422
30	*Kcnj9*	potassium inwardly-rectifying channel, subfamily J, member 9	-114	-2.4
31	*Cd300c*	CD300C molecule	-114	-14
32	*Paqr6*	progestin and adipoQ receptor family member VI	-113	-3.8
33	*Gm16538*	predicted gene 16538	-110	-25
34	*Fam151a*	family with sequence simliarity 151, member A	-108	-16
35	*Gria2*	glutamate receptor, ionotropic, AMPA2 (alpha 2)	-108	-12
36	*D630044L22Rik*	RIKEN cDNA gene D630044L22 gene	-106	-7.9
37	*Sbk2*	SH3-binding domain kinase family, member 2	-105	-1.7
38	*Gm47985*	predicted gene, 47985	-104	-3.2
39	*Hemgn*	hemogen	-103	-18
40	*Tmem178b*	transmembrane protein 178B	-98	-3.5
41	*Gm15774*	predicted gene 15774	-93	-4.0
42	*Gm10320*	SEC61 translocon subunit beta like	-93	-63
43	*Cnga1*	cyclic nucleotide gated channel alpha 1	-92	-1.6
44	*Sec14l2*	SEC14-like lipid binding 2	-92	-23
45	*Neurod4*	neurogenic differentiation 4	-90	-5.4
46	*Rgr*	retinal G protein coupled receptor	-89	-3.0
47	*A730036I17Rik*	RIKEN cDNA A730036I17 gene	-88	-10
48	*Zfp683*	zinc finger protein 683	-88	-10
49	*Gm867*	predicted gene 867	-88	-16
50	*Rhd*	Rh blood group, D antigen	-87	-17
51	*Gpr141b*	G protein-coupled receptor 141B	-87	-35
52	*Cd209a*	CD209a antigen	-86	-87
53	*A330094K24Rik*	RIKEN cDNA A330094K24 gene	-84	-19
54	*Snap25*	synaptosomal-associated protein 25	-81	-172
55	*Gfap*	glial fibrillary acidic protein	-81	-20
56	*Acot10*	acyl-CoA thioesterase 10	-78	-63
57	*Nacad*	NAC alpha domain containing	-77	-44
58	*Ugt8a*	UDP galactosyltransferase 8A	-76	-47
59	*B4galnt3*	beta-1,4-N-acetyl-galactosaminyl transferase 3	-75	1.3
60	*Klf1*	Kruppel-like transcription factor 1 (erythroid)	-74	-9.2
61	*Lrrc39*	leucine rich repeat containing 39	-72	-30
62	*Abca9*	ATP-binding cassette, sub-family A member 9	-69	-5.4
63	*Gm11690*	predicted gene 11690	-68	-8.0

64	*Cldn13*	claudin 13	-67	-14
65	*Cyp21a1*	cytochrome P450, family 21, subfamily a, polypeptide 1	-66	-4.3
66	*E030013I19Rik*	RIKEN cDNA E030013I19 gene	-64	-25
67	*Cyp4f37*	cytochrome P450, family 4, subfamily f, polypeptide 37	-63	-15
68	*Tac2*	tachykinin 2	-62	-75
69	*Rbm8a2*	RNA binding motif protein 8A2	-61	-104
70	*Ackr4*	atypical chemokine receptor 4	-61	-18
71	*Kel*	Kell blood group	-60	-7.7
72	*Edn3*	endothelin 3	-60	-8.6
73	*Tlr11*	toll-like receptor 11	-60	-12
74	*Mgst3*	microsomal glutathione S-transferase 3	-59	-14
75	*Anapc15-ps*	anaphase promoting complex C subunit 15, pseudogene	-59	-127
76	*Cfap61*	cilia and flagella associated protein 61	-59	-33
77	*Cd209d*	CD209d antigen	-58	-11
78	*Klra17*	killer cell lectin-like receptor, subfamily A, member 17	-57	-18
79	*Gm10076*	predicted gene 10076	-56	-75
80	*Apol11a*	apolipoprotein L 11a	-54	-7.9
81	*Gm13561*	predicted gene 13561	-53	-3.3
82	*Ptchd1*	patched domain containing 1	-53	-4.2
83	*Clec4b2*	C-type lectin domain family 4, member b2	-52	-31
84	*Mcf2*	mcf.2 transforming sequence	-52	-1.7
85	*Abcg4*	ATP binding cassette subfamily G member 4	-51	-12
86	*5430401H09Rik*	RIKEN cDNA 5430401H09 gene	-50	-3.5
87	*9830144P21Rik*	RIKEN cDNA 9830144P21 gene	-50	-5.9
88	*Pklr*	pyruvate kinase liver and red blood cell	-50	-6.6
89	*G6pd2*	glucose-6-phosphate dehydrogenase 2	-49	-104
90	*Icam4*	intercellular adhesion molecule 4, Landsteiner-Wiener blood group	-49	-11
91	*Gm43914*	predicted gene, 43914	-48	-27
92	*Bex6*	brain expressed family member 6	-47	-21
93	*Phyhip*	phytanoyl-CoA hydroxylase interacting protein	-47	-9.7
94	*Nags*	N-acetylglutamate synthase	-47	-4.9
95	*Btnl10*	butyrophilin-like 10	-47	-14
96	*Gm35867*	predicted gene, 35867	-47	-2.1
97	*Kcnj16*	potassium inwardly-rectifying channel, subfamily J, member 16	-46	-4.3
98	*Sptb*	spectrin beta, erythrocytic	-45	-11
99	*Chil5*	chitinase-like 5	-45	-3.7
100	*Gm17344*	predicted gene, 17344	-45	-2.5
